# Mechanistic Insights into the Neuroprotective Potential of *Aegle marmelos* (L.) Correa Fruits against Aβ-Induced Cell Toxicity in Human Neuroblastoma SH-SY5Y Cells

**DOI:** 10.3390/ph18040489

**Published:** 2025-03-28

**Authors:** Mohd Adnan, Arif Jamal Siddiqui, Fevzi Bardakci, Malvi Surti, Riadh Badraoui, Mitesh Patel

**Affiliations:** 1 Department of Biology, College of Science, University of Ha’il, Ha’il P.O. Box 2440, Saudi Arabia; drmohdadnan@gmail.com (M.A.);; 2 King Salman Center for Disability Research, Riyadh 11614, Saudi Arabia; 3 Research and Development Cell (RDC), Parul University, Waghodia, Vadodara 391760, Gujarat, India; 4 Department of Biotechnology, Parul Institute of Applied Sciences, Parul University, Waghodia, Vadodara 391760, Gujarat, India

**Keywords:** *Aegle marmelos*, neuroblastoma SH-SY5Y cells, Alzheimer’s disease, neurodisability, amyloid-β aggregation, Aβ-induced toxicity

## Abstract

**Background/Objectives**: Amyloid-β (Aβ) plaque accumulation, oxidative stress, and cholinergic dysfunction are hallmarks of Alzheimer’s disease (AD), a neurodegenerative disability that progresses over time, ultimately resulting in the loss of neurons. The side effects and limitations of current synthetic drugs have shifted attention toward natural alternatives. This study investigates the ethanolic extract of *Aegle marmelos* (L.) Corrêa fruits for their antioxidant, AChE-inhibitory, and anti-amyloidogenic properties, as well as their neuroprotective effects against amyloid beta-peptide (Aβ_1–42_). **Methods**: Phytochemical constituents were identified through HR-LCMS analysis and their antioxidant (DPPH, FRAP) and neuroprotective activities (AChE inhibition, ThT binding, MTT assay, ROS reduction, MMP restoration, and AD-related gene expression via qRT-PCR) were assessed using SHSY-5Y neuroblastoma cells. **Results**: The extract revealed the existence of flavonoids, phenols, and other bioactive substances. In vitro assays demonstrated strong antioxidant and AChE-inhibitory activities, while the ThT binding assay showed protection against amyloid-β aggregation. The extract exhibited no cytotoxicity in SHSY-5Y cells, even at a concentration of 500 μg/mL, whereas Aβ_1–42_ at 20 μM induced significant cytotoxicity. Co-treatment with Aβ_1–42_ (10 μM and 20 μM) and the extract improved cell viability (˃50%) and reduced ROS levels. Additionally, the extract restored mitochondrial membrane potential in Aβ_1–42_ treated cells, highlighting its role in preserving mitochondrial function. **Conclusions**: These findings suggest that *A. marmelos* fruits serve as a powerful source of natural antioxidants, AChE inhibitors, and anti-amyloidogenic agents, positioning them as a compelling option for AD treatment.

## 1. Introduction

Alzheimer’s disease (AD) is a prevalent neurodegenerative disorder marked by progressive memory impairment, cognitive decline, and alterations in behavior throughout its development [1]. It is well established that AD is the major disorder that develops into dementia. Millions of people around the globe suffer from this disease, and global health systems face an enormous challenge as a result [2,3]. Although decades of research have concentrated on revealing the exact etiology of AD, the causes of its onset and progression remain obscure, but it is widely accepted that genetics, environmental factors, and lifestyle factors are all associated with the emergence and advancement of AD [4,5]. Aside from the build-up of amyloid-β peptides, a key feature of AD is the creation of amyloid-β plaque in the brain [6,7]. As a result of these plaques, neuronal communication is disrupted, inflammatory responses are triggered, and neuronal death is caused. Being one of the pathological characteristics of AD, neurofibrillary tangles are formed as a result of hyperphosphorylated tau protein and act as further signs and symptoms of the disease [8,9]. These tangles worsen neuronal dysfunction and are a major driver in the progression of the disease [10]. The amyloid cascade hypothesis proposes that amyloid peptide deposition is a key factor in AD pathogenesis, triggering multiple neurotoxic events such as oxidative stress, mitochondrial dysfunction, and synaptic loss [11].

Oxidative stress is a major contributing aspect in the pathophysiology of AD. As the brain contains a high content of monounsaturated fatty acids and is highly metabolic, it is prone to oxidative damage [12]. Aβ peptides can enhance the synthesis of ROS, which subsequently harms cellular components like lipids, proteins, and DNA. This oxidative damage impairs neuronal activity and contributes to the neurodegenerative process [13]. Moreover, the mitochondria, the powerhouse of the cell, are also adversely affected by Aβ peptides. Mitochondrial dysfunction, resulting from impaired energy metabolism and elevated ROS production, intensifies neuronal damage and promotes apoptosis [14].

Current therapeutic strategies for AD mainly aim at improving symptoms instead of addressing the fundamental mechanisms of the disease [15]. The current drug treatment methods, which include N-methyl-D-aspartate (NMDA) receptor antagonists and acetylcholinesterase inhibitors, have numerous negative side effects in addition to their limited therapeutic benefits [16]. Therefore, it is imperative that new therapeutic agents are developed that can successfully target the diverse pathological processes that promote AD. Natural compounds derived from medicinal plants have gained increased attention recently as methods to promote neuroprotection [17,18]. There are a wide range of biological activities associated with these compounds, such as antioxidant, anti-inflammatory, and anti-apoptotic effects [19,20]. Among the various plants studied, *A. marmelos*, often called Bael, has historically been utilized in Ayurvedic medicine for its diverse therapeutic properties. According to studies, the fruit of *A. marmelos* contains a rich content of phytochemical constituents like phenols, flavonoids, coumarins, terpenoids, etc., which are capable of exhibiting potent antioxidant and neuroprotective properties, as well as being a potential food source [21,22].

An important group of polyphenols found in *A. marmelos* are flavonoids, which scavenge free radicals and chelate metal ions, helping to combat oxidative stress [23,24]. Additionally, flavonoids can modulate metabolic pathways that are important for cell survival and apoptosis, providing a protective effect against various neurotoxic damage [25,26]. Coumarins and terpenoids, also found in *A. marmelos*, reportedly possess neuroprotective and anti-inflammatory activities. In addition, these compounds prevent AD from progressing by reducing the release of pro-inflammatory cytokines and microglial activation, which cooperate to cause neuroinflammation, a key contributor to this disease [27,28].

The neuroprotective potential of *A. marmelos* fruits and other parts have been explored in a few studies. Several studies have demonstrated that the natural extracts are capable of protecting neuronal cells from oxidative stress, reducing neuroinflammation, and enhancing cognitive function in animal models of neurodegeneration, with the conclusions that the extracts are beneficial to neuronal cells [27,28]. However, the mechanism responsible for *A. marmelos* fruits’ neuroprotective actions, particularly against Aβ peptide-induced toxicity, remain to be entirely revealed. In view of this, the present study was undertaken to evaluate whether AMFE possesses any neuroprotective properties towards toxicity induced by Aβ peptides in human neuroblastoma SH-SY5Y cells. This cell line is commonly utilized as an in vitro model for studying neurodegenerative processes and drug screening, as it possesses many of the biochemical and morphological characteristics of mature neurons [28,29,30]. The hypothesis was that the possible presence of several bioactive compounds in AMFE may alleviate Aβ-derived neurotoxicity by improving cell viability, reducing damage caused by ROS, and maintaining the activity of mitochondria. For the purpose of testing this hypothesis, a treatment of SH-SY5Y cells was carried out with Aβ peptides, both when AMFE was present or absent. The cytoprotective effects of the extract on cellular survival were assessed using the MTT assay, which detects the metabolic function of cells as an indicator of cell health. Additionally, the antioxidant activity of the extract was determined by the content of intracellular ROS and the effect of the extract on mitochondrial function by analyzing the mitochondrial membrane potential.

Moreover, currently, AD remains a significant public health challenge due to the lack of effective disease-modifying treatments. Current pharmacological interventions including cholinesterase inhibitors and NMDA receptor antagonists provide only symptomatic relief and fail to halt disease progression [16]. Emerging evidence suggests that oxidative stress, mitochondrial dysfunction, and cholinergic deficits play critical roles in AD pathogenesis, highlighting the need for therapeutic agents that target multiple pathological pathways [14]. Despite the growing interest in plant-derived bioactive compounds with neuroprotective properties, limited studies have explored the potential of AMFE in mitigating Aβ-induced toxicity. Given its antioxidant, anti-inflammatory, and acetylcholinesterase (AChE) inhibitory properties, AMFE presents a promising candidate for AD intervention [27,28]. However, the precise mechanisms by which AMFE exerts its neuroprotective effects remain poorly understood. This study aims to address this gap by evaluating the multifaceted protective effects of AMFE against Aβ_1–42_ induced toxicity in SH-SY5Y cells, thereby advancing the current knowledge on potential natural therapeutic approaches to AD management.

## 2. Results

### 2.1. Phytochemical Screening and Content Analysis

As a result of the primary phytochemical analysis of AMFE, alkaloids, saponins, terpenoids, flavonoids, and phenolic compounds were detected. Previous studies indicate that there is an inherent relationship between flavonoid content and phenolic content and antioxidant activity. These natural compounds are known to mitigate the progression of neurological diseases in both in vitro and in vivo assays. The overall contents of phenolic and flavonoid compounds in the AMFE were quantified via calibration curves for gallic acid and quercetin, respectively. The phenol content in the dry extract was 32.47 mg/mL gallic acid equivalents, while the content of flavonoid content was 20.59 mg/mL quercetin equivalents.

### 2.2. UV-VIS Analysis

**Figure 1:**
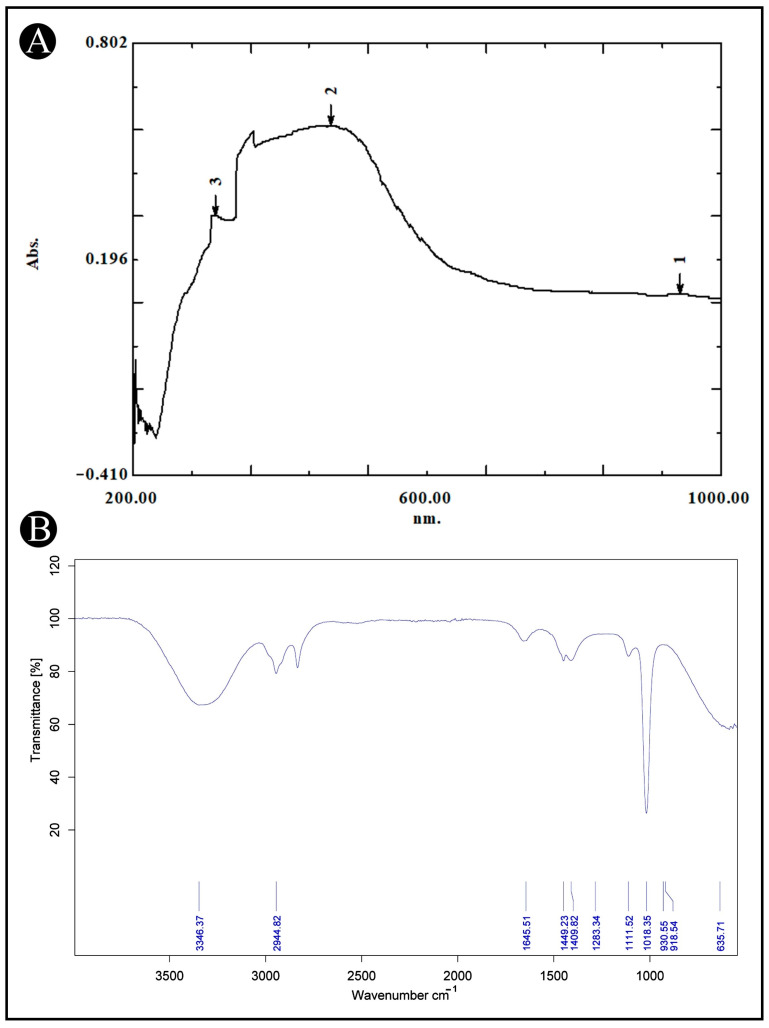
UV-VIS and FT-IR analysis of AMFE. (**A**) The UV-VIS spectrum shows the absorption peaks of AMFE, indicating the presence of specific bioactive compounds within the extract. (**B**) The FT-IR spectrum displays characteristic functional groups present in AMFE.

The UV-VIS spectral analysis of AMFE was conducted across a wavelength range of 200–1000 nm (Figure 1A). The analysis revealed the probability of the presence of different phytoconstituents based on their absorption peaks. Flavonoids and their derivatives exhibit distinct absorption peaks within the range of 200 and 400 nm, with prominent maxima occurring within the 230–285 nm and 300–350 nm ranges. Moreover, alkaloids, flavanols, flavonoids, and phenolic acids are detectable across a broader absorption spectrum, extending from 270 to 670 nm. These findings indicate the presence of a range of phytochemicals in AMFE.

### 2.3. FTIR Analysis

AMFE was analyzed using FTIR to determine its functional groups and chemical components. The FTIR spectra, shown in (Figure 1B), reveal bands within the range of 4000–500 cm^−1^. These bands correspond to various functional groups, including O-H, indicative of alcohols and phenols. The results confirm the presence of several functional groups such as, 635.71 cm^−1^ for C-I (haloalkane), 1018.35–1283.34 cm^−1^ for C-F (haloalkane), 1409.82–1449.23 cm^−1^ for C-O or C-F (alcohols, ethers, esters and haloalkane), 1645.51 cm^−1^ for C=O (saturated acid), 2944.82 cm^−1^ for C-H (aromatic), and 33,362.37 cm^−1^ for O-H (alcohol and phenols). These groups are characteristic of plant secondary metabolites.

### 2.4. LC-MS Analysis of AMFE

**Figure 2:**
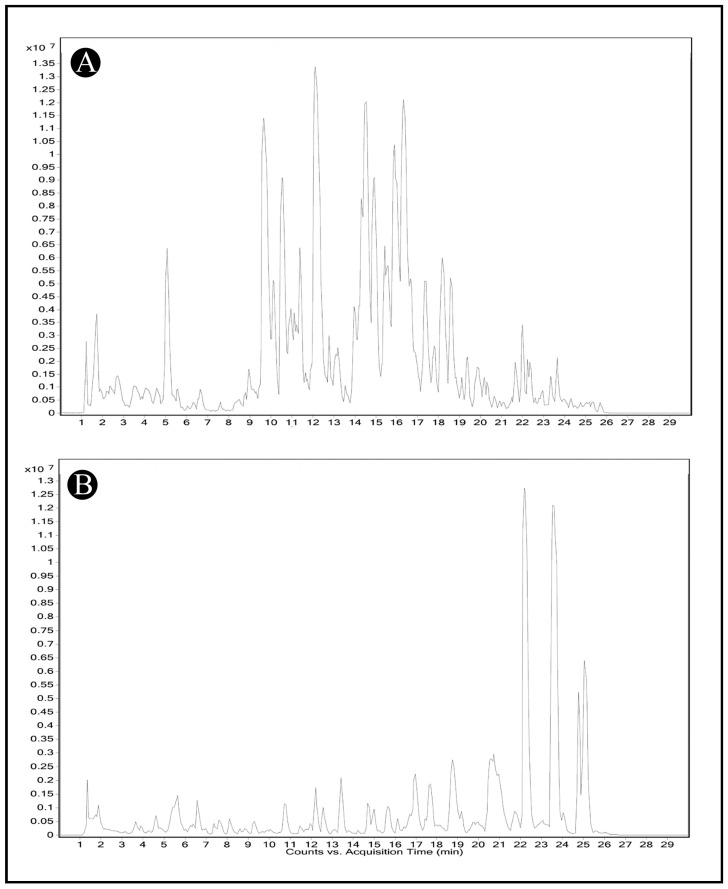
Identification of phytochemical constituents from AMFE via HR-LCMS analysis. (**A**) Chromatogram obtained in positive mode of analysis. (**B**) Chromatogram obtained in negative mode of analysis.

The LC-MS analysis of AMFE, as depicted in (Figure 2A,B), revealed multiple peaks corresponding to various phytochemical constituents. The comparison of these masses with the Mass Bank library confirmed the presence of different classes of compounds listed in (Table 1 and Table 2).

### 2.5. Antioxidant Activity

**Figure 3:**
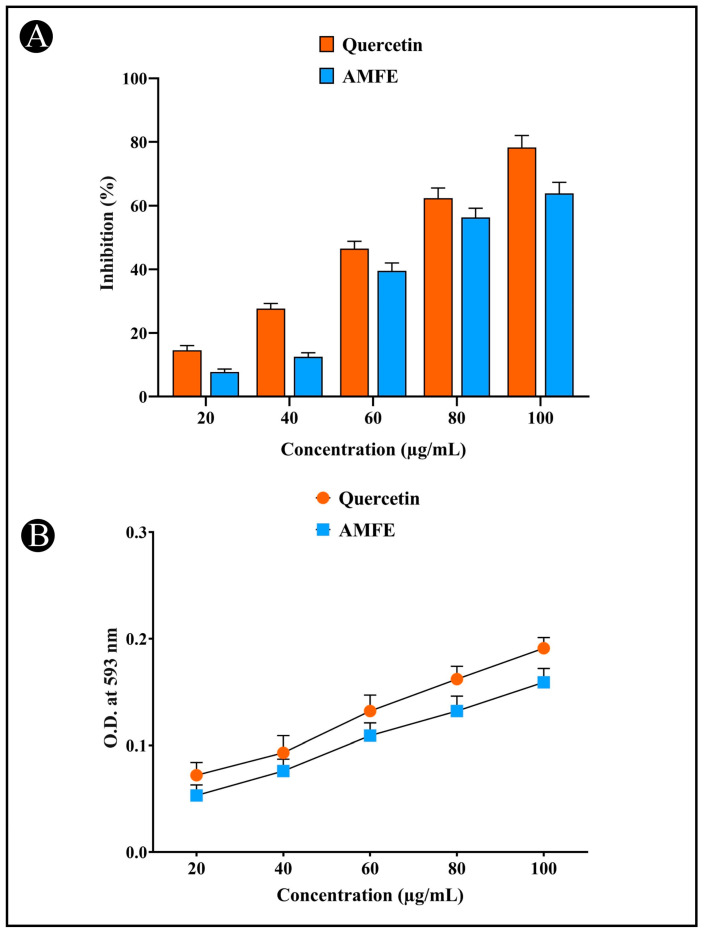
Determination of antioxidant potential of AMFE. (**A**) DPPH (2,2-diphenyl-1-picrylhydrazyl) radical scavenging activity. (**B**) Ferric reducing antioxidant power (FRAP) assay.

Oxidative stress markers are recognized for their role in triggering neurodegeneration, resulting in cellular damage and death. To assess antioxidant activity, both the DPPH and FRAP assays were utilized. The DPPH assay detects free radicals by measuring absorbance at 515 nm, while the FRAP assay measures absorbance at 593 nm. A notable color change from deep violet to pale yellow in the DPPH assay indicates antioxidant activity, while the FRAP assay showed an increase in the Fe^2+^-TPTZ complex, suggesting effective electron donation by the extracts. The AMFE demonstrated concentration-dependent antioxidant activity. This activity was comparable to that of quercetin, a known antioxidant. The results indicate that AMFE is effective in scavenging free radicals and donating electrons, contributing to their potent antioxidant properties (Figure 3A,B).

### 2.6. Inhibition of Acetylcholinesterase (AChE) and the Potential for Thioflavin T (ThT) Binding

**Figure 4:**
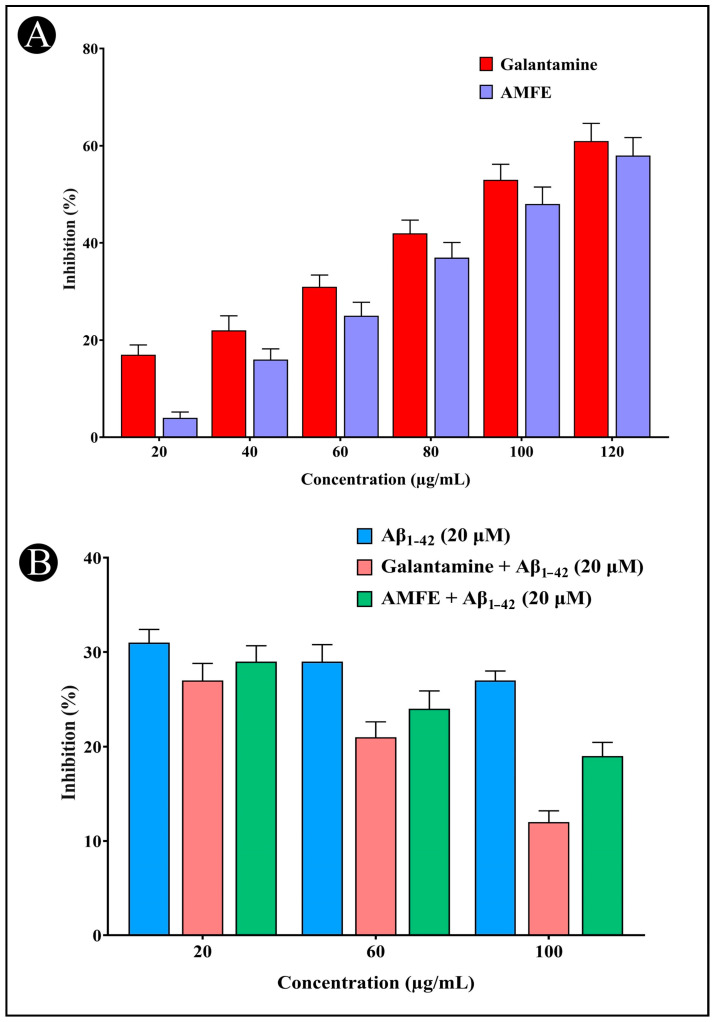
Assessment of AChE inhibition and amyloid aggregation using the Thioflavin-T assay. (**A**) AChE inhibition activity of different concentrations of AMFE and positive control (galantamine). (**B**) Results of the Thioflavin-T assay activity of Aβ_1–42_, different concentrations of AMFE and positive control (galantamine).

The AChE inhibitory activity of AMFE is illustrated in (Figure 4A). The concentration-dependent inhibition (20, 40, 60, 80, 100, and 120 μg/mL) of AChE by AMFE was demonstrated. A number of phytochemicals, including phenolic and flavonoid compounds, have been shown to have neuroprotective properties as they are found to inhibit AChE activity, which represents an effective strategy for treating AD. The neuroprotective potential of AMFE was further evaluated using the ThT fluorescence assay (Figure 4B). The results demonstrate that AMFE inhibited the binding of ThT to amyloid, indicating its effectiveness in preventing amyloid-β aggregation.

### 2.7. MTT Assay and Cell Viability

**Figure 5:**
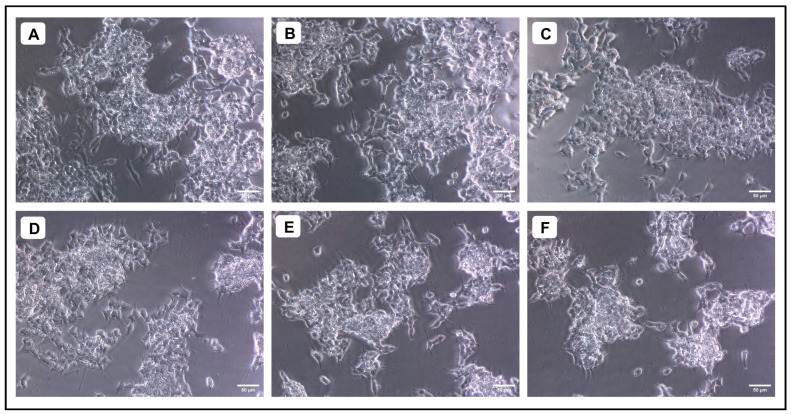
Evaluation of the cytotoxicity of different concentrations of *A. marmelos* fruit extract against SH-SY5Y cells; (**A**) 15.625 (µg/mL), (**B**) 31.25 (µg/mL), (**C**) 62.5 (µg/mL), (**D**) 125 (µg/mL), (**E**) 250 (µg/mL), and (**F**) 500 (µg/mL).

**Figure 6:**
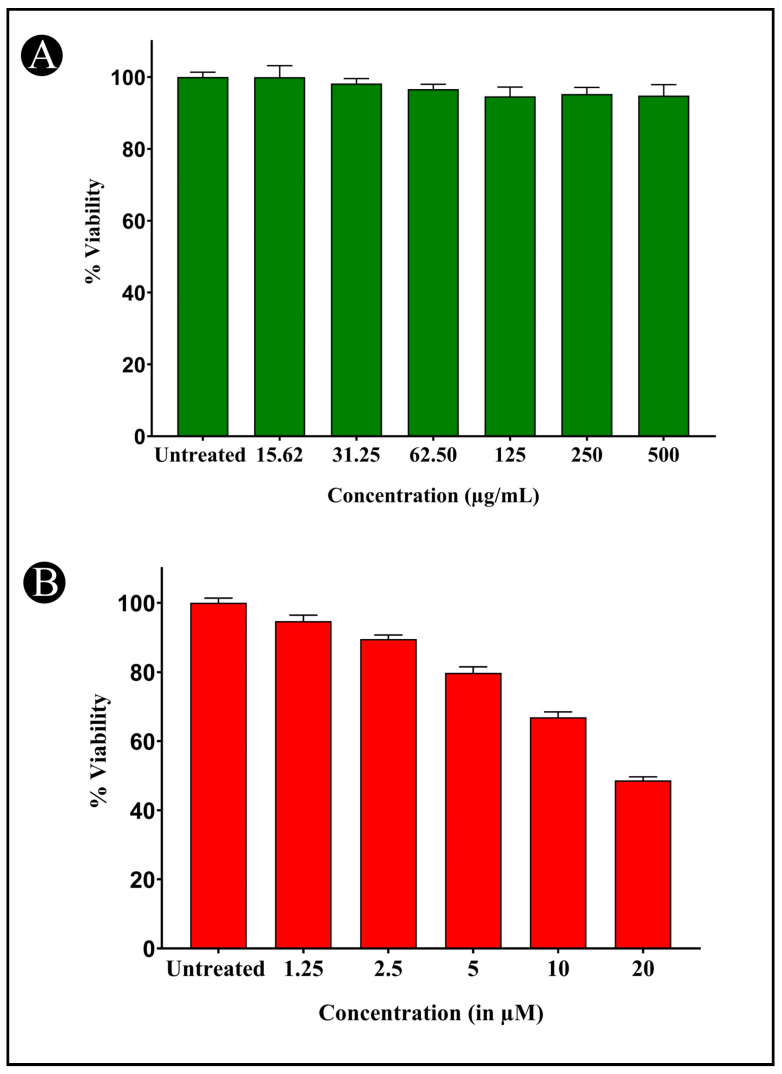
Cytotoxicity assessment of AMFE and Aβ1-42 against SH-SY5Y cells using the MTT assay. (**A**) Cell viability (%) of SH-SY5Y cells exposed to various concentrations of AMFE for 24 h. (**B**) Cell viability (%) of SH-SY5Y cells exposed to different concentrations of Aβ_1–42_ for 24 h.

**Figure 7:**
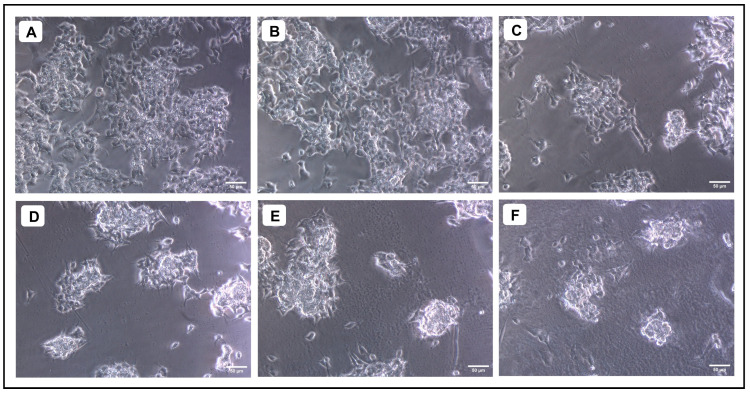
Evaluation of the cytotoxicity of different concentrations of Aβ_1–42_ against SH-SY5Y cells. (**A**) Untreated, (**B**) 1.25 µM, (**C**) 2.5 µM, (**D**) 5 µM (**E**) 10 µM, (**F**) 20 µM.

**Figure 8:**
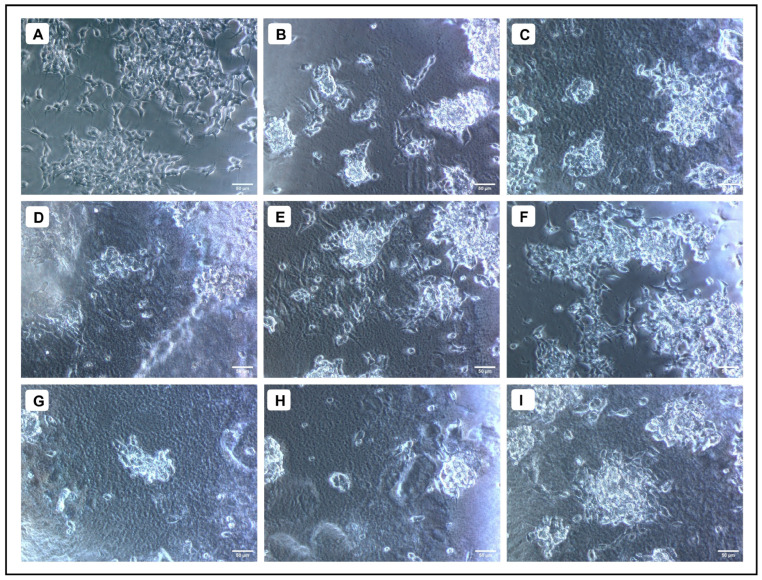
Neuroprotective effect of AMFE against Aβ_1–42_-induced cytotoxicity in SH-SY5Y cells. (**A**) Untreated cells, (**B**) Aβ_1–42_ (10 µM), (**C**) Aβ_1–42_ (20 µM), (**D**) Aβ_1–42_ (10 µM) + AMFE (15.62 µg/mL) (**E**) Aβ_1–42_ (10 µM) + AMFE (31.25 µg/mL), (**F**) Aβ_1–42_ (10 µM) + AMFE (62.50 µg/mL), (**G**) Aβ_1–42_ (20 µM) + AMFE (15.62 µg/mL), (**H**) Aβ_1–42_ (20 µM) + AMFE (31.25 µg/mL), (**I**) Aβ_1–42_ (20 µM) + AMFE (62.50 µg/mL).

When various concentrations of AMFE (15.62, 31.25, 62.5, 125, 250 and 500 μg/mL) were evaluated for their cytotoxicity against SH-SY5Y cells, no significant changes were observed in cell viability or cell morphology. Even at very high concentrations (500 μg/mL), only a slight reduction in cell viability was noted, indicating that AMFE exhibits minimal cytotoxicity towards SH-SY5Y cells (Figure 5A–F and Figure 6A). Treatment with Aβ_1–42_ at varying concentrations (1.25, 2.5, 5, 10 and 20 μM) exhibited different levels of cytotoxicity on SH-SY5Y cells. The IC_50_ concentration of Aβ_1–42_ for SH-SY5Y cells was found to be 18.54 µM. Specifically, Aβ_1–42_ at 20 μM caused significant cytotoxicity, leading to changes such as cell loss, shrinkage, and altered cell morphology, as observed through microscopic examination (Figure 6B and Figure 7A–F). Whereas, as a result of exposure to Aβ_1–42_ in SHSY-5Y cells (10 μM and 20 μM) with different concentrations of AMFE (15.62, 31.25, 62.5 μg/mL) for 24 h, the MTT assay was used to assess cell survival. Groups exposed to amyloid beta demonstrated that the viability of cells was significantly reduced, with toxicity escalating in a dose-dependent way. However, the administration of different concentrations of AMFE in conjugation with Aβ_1–42_ (10 and 20 μM) for 24 h markedly enhanced cell viability compared to the groups treated solely with Aβ_1–42_ (10 and 20 μM), thereby confirming its neuroprotective properties (Figure 8A–I).

### 2.8. Effect of AMFE on ROS Production

**Figure 9:**
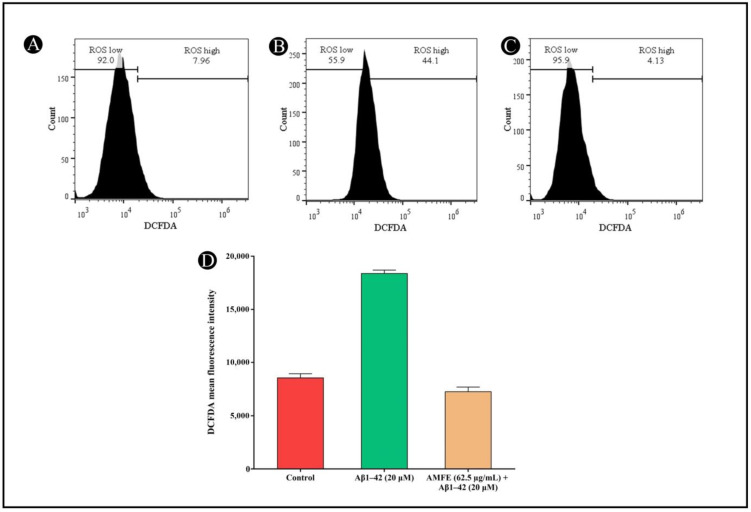
Measurement of intracellular ROS levels in SH-SY5Y cells using H_2_DCFDA dye via flow cytometry. (**A**) Untreated cells showing baseline ROS levels. (**B**) SH-SY5Y cells treated with Aβ_1–42_ (20 µM) showing elevated ROS levels. (**C**) SH-SY5Y cells pre-treated with AMFE (62.50 µg/mL) followed by Aβ_1–42_ (20 µM), showing reduced ROS levels. (**D**) Quantitative analysis of H_2_DCFDA fluorescence intensity for each treatment group, indicating the effects of AMFE on reducing ROS generation induced by Aβ_1–42_.

It is widely recognized that oxidative stress induced by Aβ plays a pivotal role in the progression of AD. Oxidative stress not only stimulates the generation of amyloid-β but also exacerbates the pathology. In the present study, ROS generation was measured via H_2_DCFDA staining, which fluoresces upon reacting with ROS. SH-SY5Y cells were exposed to 20 μM Aβ_1–42_, both with and without AMFE, for 24 h. The findings demonstrated a substantial reduction in ROS levels in the groups treated with Aβ_1–42_ (20 μM) combined with AMFE at concentrations of 62.5 μg/mL. The obtained results suggest that AMFE effectively prevented Aβ_1–42_-induced ROS production, as demonstrated by the remarkable reduction in ROS levels in the treated groups (Figure 9A–D). These results were obtained using flow cytometry, where an increase in cellular ROS generation corresponded with an increase in H_2_DCFDA fluorescence intensity. The AMFE inhibited Aβ_1–42_ (20 μM) derived toxicity, as detected via a reduction in fluorescent intensity, underscoring the potential of AMFE in suppressing ROS production.

### 2.9. Effects of AMFE on MMP

**Figure 10:**
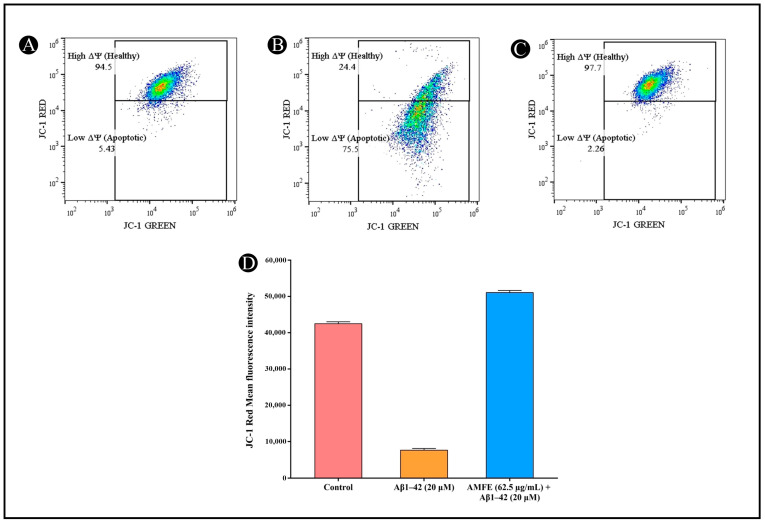
Measurement of mitochondrial membrane potential (Δψ) in SH-SY5Y cells using JC-1 staining and flow cytometry. (**A**) Untreated cells showing intact mitochondrial membrane potential. (**B**) SH-SY5Y cells treated with Aβ_1–42_ (20 µM), showing depolarized mitochondrial membrane potential. (**C**) SH-SY5Y cells pre-treated with AMFE (62.50 µg/mL) followed by Aβ_1–42_ (20 µM), showing partial restoration of mitochondrial membrane potential. (**D**) Quantitative analysis of JC-1 red fluorescence intensity, representing mitochondrial membrane integrity for each treatment group.

The MMP (Δψ) in SH-SY5Y cells was analyzed via the JC-1 dye after pre-treatment with AMFE, followed by Aβ_1–42_ (20 μM) exposure. Cells pre-treated with AMFE (62.5 μg/mL) displayed a substantial rise in healthy cells exhibiting a high mitochondrial membrane potential (Δψ), compared to the group treated solely with β-Amyloid. Within Aβ_1–42_-treated cells, a notable increase in cells with depolarized Δψ was observed, indicating mitochondrial dysfunction. This depolarization was evidenced by a reduction in JC-1 red fluorescence, corresponding to a reduction in healthy mitochondrial activity. A significant reduction within the mean fluorescence intensity (MFI) of red fluorescence was noted, along with an increased population of cells in the apoptotic gate, indicating low red fluorescence and higher mitochondrial depolarization. In contrast, AMFE pre-treated cells demonstrated a restoration of MMP, reflected by an enhancement in red fluorescence. JC-1 green fluorescence (indicative of depolarized mitochondria) was collected using the FL1 detector (525 nm bandpass filter), while JC-1 red fluorescence (indicative of polarized mitochondria) was collected using the FL3 detector (620 nm bandpass filter). These results suggest that AMFE pre-treatment mitigates mitochondrial depolarization induced by Aβ_1–42_, enhancing cell survival and maintaining mitochondrial function (Figure 10A–D).

### 2.10. Impact of AMFE on Expression of AD-Related Genes

**Figure 11:**
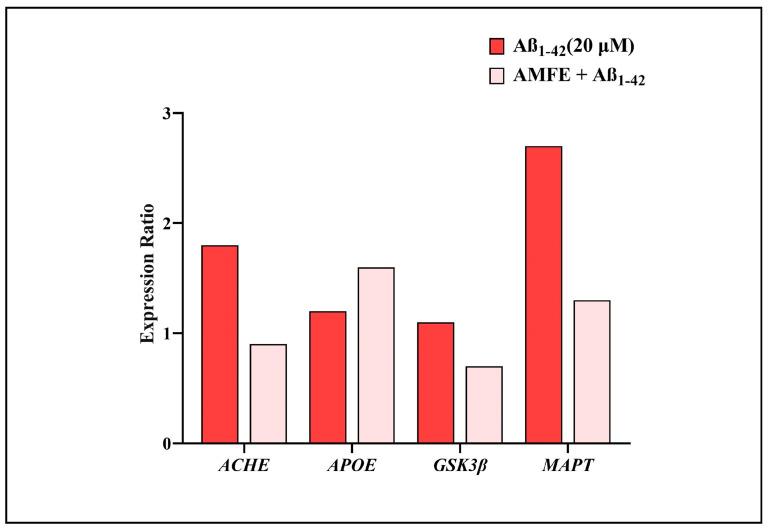
Impact of AMFE on the mRNA expression of genes associated with AD. mRNA levels were quantified using RT-qPCR, with results presented as expression ratios normalized to reference genes.

The mRNA expression levels of several key genes (ACHE, APOE, GSK3β, and MAPT) associated with AD were examined to assess the neuroprotective effects of AMFE. Figure 11 shows that SH-SY5Y cells treated with Aβ_1–42_ showed an upregulation in the mRNA expression of the ACHE, GSK3β, and MAPT genes (which encode the microtubule-associated protein tau) and a decrease in the APOE, relative to the untreated control group. Pre-treating the cells with 62.5 µg/mL of AMFE for 30 min effectively inhibited the overexpression of ACHE, GSK3β, and MAPT genes and enhanced the expression of APOE gene. The MAPT gene is responsible for producing tau protein, which, when hyperphosphorylated, becomes the primary component of neurofibrillary tangles, structures implicated in the advancement of AD. The hyperphosphorylation of tau is recognized as a key aspect in Aβ-induced neurodegeneration. In this study, it was found that AMFE could provide neuroprotection to SH-SY5Y cells by preventing overexpression of MAPT, potentially reducing the development of neurofibrillary tangles that contribute to oxidative stress, cell damage, and subsequent neurodegeneration. Further research, such as proteomic analyses, are required to validate these results.

## 3. Discussion

A primary contributing factor to dementia, AD presents with the following characteristics, such as a progressive deterioration of cognitive functions, memory impairment, and neuropathological traits, including the build-up of Aβ-plaques, neurofibrillary tangles, synaptic dysfunction, and neuronal degeneration [31]. The accretion of Aβ plaques is crucial in triggering a series of neurotoxic actions that eventually result in neuronal degeneration in the brain [32]. One of the primary pathways through which Aβ exerts its neurotoxicity is by inducing oxidative stress and the impairment of mitochondrial function, which in turn result in neuronal death and cognitive deficits [33]. Additionally, cholinergic dysfunction, marked by increased acetylcholinesterase (AChE) activity, contributes significantly to the memory loss observed in AD patients [34]. Given the multifaceted pathogenesis of AD, targeting multiple pathways involved in Aβ-induced neurotoxicity may present an effective treatment strategy. In the present study, to induce neurotoxicity in the SH-SY5Y neuroblastoma cells, 10 and 20 µM concentrations of Aβ_1–42_ were utilized. This selection was based on a thorough review of the existing literature, which has shown these concentrations to effectively model Aβ_1–42_ toxicity in this cell line. These concentrations are frequently employed to induce significant, yet sub-lethal, neuronal damage, mimicking the early stages of amyloid plaque formation in AD [35,36]. Specifically, numerous studies have demonstrated that within this range, Aβ_1–42_ induces measurable reductions in cell viability, increases in oxidative stress, and disruptions in mitochondrial function, consistent with the pathological hallmarks of AD. Furthermore, these concentrations allow for the observation of the dose-dependent effects of potential neuroprotective agents, facilitating a clear assessment of their efficacy [37,38]. This range was opted for in the present study rather than lower concentrations because while lower concentrations may be more physiologically relevant to the very early stages of AD, they often do not produce a strong and quantifiable level of cellular stress in the in vitro model, which is necessary to properly evaluate the effect of a neuroprotective substance. As part of the current investigation, the neuroprotective potential of AMFE (15.62 to 500 µg/mL) was evaluated towards Aβ_1–42_ cytotoxicity in SH-SY5Y cells, focusing on AChE inhibition, ROS generation, and MMP disruption. The AMFE dose range was determined through a preliminary series of experiments to establish a non-toxic concentration range for SH-SY5Y cells. A broader range of concentrations were initially tested, which resulted in those concentrations above 500 µg/mL exhibiting significant cytotoxicity towards SH-SY5Y cells. Therefore, a concentration of 15.62 to 500 µg/mL was used to ensure that the observed neuroprotective effects were not confounded by inherent toxicity. This range also aligns with a previous study investigating the neuroprotective potential of AMFE against Aβ toxicity in a transgenic *Caenorhabditis elegans* [30]. The results of the current study reveal that AMFE exerts significant neuroprotective effects, indicating its promise in AD treatment options.

The cholinergic system is vital for learning and memory, and its impairment is among the initial indicators of AD [34]. Acetylcholinesterase (AChE) is an enzyme that is responsible for converting acetylcholine into a neurotransmitter necessary for cognition. Elevated AChE activity leads to reduced acetylcholine levels, contributing to the cognitive deficits characteristic of AD [39]. Current pharmacological therapies for AD include medications like donepezil and rivastigmine and aim to inhibit AChE activity, thereby increasing acetylcholine availability and improving cognitive function. However, these treatments only offer symptomatic relief and are associated with various side effects, highlighting the need for safer and more effective alternatives [40,41]. In this study, we demonstrated that AMFE significantly inhibits AChE activity in SH-SY5Y cells. This finding is particularly important because it suggests that AMFE has the potential to enhance cholinergic neurotransmission by preserving acetylcholine levels in the brain. Moreover, the natural origin of *A. marmelos* fruits could offer a safer alternative to synthetic AChE inhibitors, with fewer side effects. The AChE inhibitory activity observed in the current study aligns with published studies on the neuroprotective potential of plant-derived compounds, particularly those rich in phenolic and flavonoid content [27,40,42]. There have been many studies highlighting the importance of plant-based antioxidants in modulating cholinergic function and protecting neurons from Aβ-induced toxicity [43,44,45]. The ability of AMFE to inhibit AChE activity underscores its promise, presenting it as a therapeutic approach for AD by addressing cholinergic dysfunction.

It is well known that oxidative stress plays a crucial role in the development of AD. Numerous studies have demonstrated that the Aβ peptide facilitates the production of ROS, resulting in oxidative harm to lipids, proteins, and DNA [13,46,47]. Activated ROS induce cellular damage by being highly reactive molecules. They are thought to contribute to the progression of various neurodegenerative diseases, including AD [13]. The brain exhibits a specific susceptibility to oxidative stress because of its abundance of lipids and high oxygen consumption. The increase in ROS disrupts neuronal homeostasis, resulting in mitochondrial dysfunction, activation of apoptotic pathways, and, ultimately, neuronal death [48,49]. In the current study, exposure to Aβ_1–42_ in SH-SY5Y cells resulted in a marked elevation of ROS levels, confirming the oxidative stress-inducing potential of Aβ. However, co-treatment with AMFE significantly reduced ROS levels in the cells, indicating its potent antioxidant activity. The antioxidant properties of *A. marmelos* can be ascribed to the abundance of phytoconstituents, including flavonoids and phenolic compounds, that are known to effectively neutralize free radicals and alleviate oxidative stress in a variety of models [23]. Previous studies have reported that plant-based antioxidants can effectively reduce ROS production and protect neurons from Aβ-induced oxidative damage [50,51]. The reduction in ROS levels observed in this study suggests that AMFE may act by neutralizing free radicals and preventing oxidative damage, thereby protecting neurons from Aβ-induced cytotoxicity. The capacity to diminish oxidative stress is crucial for neuroprotection, given that oxidative damage is a major factor in the neurodegenerative processes detected in AD [50,52,53]. Thus, the antioxidant activity of AMFE further supports the possibility of using it as a therapy for AD.

Cellular energy is generated by mitochondria, which are essential in contributing to neuronal function and health. However, mitochondria are also a major target of oxidative stress and their dysfunction is closely linked to neurodegenerative diseases, including AD [54,55]. A key early indicator of mitochondrial dysfunction in AD is the reduction in MMP, which is essential for sustaining mitochondrial activity and ATP synthesis. The loss of MMP leads towards the activation and release of pro-apoptotic factors, activation of caspases, and, ultimately, neuronal apoptosis [56]. In the current investigation, treatment through Aβ_1–42_ resulted in a notable decrease in MMP in SH-SY5Y cells, suggesting mitochondrial dysfunction. However, co-treatment with AMFE effectively restored MMP, suggesting that the extract mitigates mitochondrial damage and preserves mitochondrial function. The restoration of MMP is indicative of the protective effects of AMFE on mitochondrial integrity, which is necessary to maintain cellular energy metabolism and prevent apoptosis. The ability of AMFE to restore MMP may be linked to its antioxidant properties, as oxidative stress is a major contributor to mitochondrial dysfunction [13,46]. By reducing ROS levels, AMFE may protect mitochondria from oxidative damage, thereby preserving MMP and preventing the activation of apoptotic pathways. Several studies have reported similar findings, where plant-derived compounds were able to restore MMP and protect neurons from Aβ-induced mitochondrial dysfunction [57,58].

Furthermore, the neuroprotective potential of AMFE was further confirmed by analyzing the mRNA expression of ACHE, APOE, GSK3β, and MAPT genes linked to AD. The obtained results show that SH-SY5Y cells exposed to Aβ_1–42_ exhibited elevated mRNA levels in contrast to the control group. Notably, pre-treatment with AMFE at a very low concentration significantly suppressed the overexpression of these genes. The MAPT gene, which encodes the tau protein, is crucial in the progression of AD, as hyperphosphorylated tau forms the core of neurofibrillary tangles [59]. These tangles are closely associated with Aβ-induced neurodegeneration [60]. The results suggest that AMFE exerts a neuroprotective effect by mitigating the overexpression of MAPT, thereby potentially limiting the development of neurofibrillary tangles, oxidative stress, and subsequent neuronal damage. However, additional studies, such as proteomic analyses, are necessary to further corroborate these findings.

Overall, the neuroprotective potential of AMFE demonstrated in this study is multifaceted, primarily driven by the presence of flavonoids, phenols, and other bioactive compounds targeting several key pathways involved in Aβ-induced neurotoxicity. The ability to inhibit AChE activity suggests that AMFE may enhance cholinergic neurotransmission and improve cognitive function in AD. AChE inhibition prolongs the availability of acetylcholine at the synapses, which is essential for maintaining cognitive function in AD. Furthermore, AChE has been implicated in promoting Aβ aggregation and plaque formation [61]. By inhibiting AChE activity, AMFE not only supports cholinergic function but also indirectly reduces the aggregation of amyloid fibrils, further contributing to neuroprotection. The results of the ThT binding assay confirm that AMFE prevents Aβ aggregation, indicating its ability to interfere with the amyloidogenic cascade and reduce the formation of toxic oligomers and fibrils, which are known to trigger neurodegenerative processes in AD [62,63]. Apart from this, one of the key mechanisms involves the strong antioxidant activity of AMFE, which effectively neutralizes the excessive ROS generated due to Aβ_1–42_ toxicity [64]. Elevated ROS levels lead to oxidative stress, lipid peroxidation, and mitochondrial dysfunction, culminating in neuronal apoptosis [65,66,67]. By scavenging ROS and reducing oxidative stress, AMFE prevents mitochondrial damage and preserves cellular homeostasis, which is reflected in the restoration of MMP in Aβ_1–42_-treated cells. This mitochondrial protection is likely mediated by the inhibition of mitochondrial permeability transition pore opening, preventing the release of pro-apoptotic factors and maintaining cellular energy production [68,69]. This preservation of mitochondrial integrity protects against caspase activation and subsequent apoptosis, contributing to improved cell viability observed in co-treated cells. The ability of AMFE to significantly improve cell viability under Aβ-induced cytotoxicity conditions determines its potential as a potent neuroprotective agent. There is consistency between these findings and previous research on the neuroprotective effects of plant-derived compounds, particularly those rich in antioxidants [42,51,70]. Research has been conducted extensively on the potential of natural products to prevent or treat neurodegenerative diseases, and many plant-based compounds have shown promise in protecting neurons from Aβ-induced toxicity [18,71]. AMFE, with its diverse range of bioactive compounds, contributes towards the increasing research findings that supports the utilization of natural antioxidants as potential therapeutics for AD. Moreover, it is noteworthy that dysfunctions in both neurons and oligodendrocytes contribute to the progression of several neurodegenerative diseases [72]. Given the multifaceted neuroprotective effects of AMFE, future investigations should explore its potential impact on oligodendrocyte function and myelination, which may further enhance its therapeutic relevance in neurodegenerative disorders.

Although the findings of this study are encouraging, additional research is necessary to thoroughly clarify the mechanisms that contribute to the protective effects on the neuronal health of AMFE. Future studies should concentrate on identifying the exact bioactive molecules that account for the observed effects and identifying their exact molecular targets. Furthermore, animal studies and clinical trials are essential to evaluate the safety, efficacy, and pharmacokinetics of AMFE in humans. The development of natural substances as treatment options for AD holds significant potential, as they offer a safer alternative to synthetic drugs and may provide a more comprehensive approach to targeting the complex nature of the condition. AMFE stands out as a promising option for further advancement, and its neuroprotective properties warrant continued investigation.

## 4. Materials and Methods

### 4.1. Collection and Extract Preparation

The plant material was sourced from the local market. Further, 50 g of fruit powder and 500 mL of 85% ethanol were combined to yield AMFE, which was then continuously shaken at 110 rpm for 48 h at 37 °C. The ethanol phase was filtered using Whatman No. 1 filter paper, and the filtrate was then concentrated using a rotary evaporator (Rotavapor^®^ R-300, Büchi Labortechnik AG, Flawil, Switzerland) to yield a dry residue. The dried extract was kept in brown bottles at room temperature to ensure its reliability and safety [73].

### 4.2. Screening for Phytochemicals

The AMFE was subjected to a phytochemical screening to identify various compounds, including flavonoids, saponins, terpenoids, alkaloids, phenolic compounds, and others. The screening was conducted following the method previously described by Pratap and Shantaram (2022) [74].

### 4.3. UV-VIS and FTIR Analysis

The functional groups present in AMFE were identified using UV-VIS analysis (UV-2600, Shimadzu, Kyoto, Japan) and FTIR analysis (Bruker^®^, Billerica, MA, USA). A wavelength between 200 and 1000 nm was scanned at a resolution of 1 nm for UV-VIS analysis, whereas in FTIR, spectra were scanned within the 4000–500 cm−^1^ range with 32 scans and a resolution of 4 cm^−1^. A comparison of the intensity bands resulting from the analysis was then carried out with standard reference values to identify the functional groups [74].

### 4.4. Estimation of Total Flavonoids

The assay was carried out using a colorimetric method to determine the total flavonoid content in the AMFE, as mentioned by Sugimoto et al. (2000) [75]. To prepare the solution, 200 µL of AMFE (1 mg/mL) and 0.5 mL of 2% AlCl_3_ solution were combined in a clean test tube. Incubation was enabled at 37 °C for 1 h before absorbance at 510 nm was taken. For the analysis of total flavonoids, quercetin (1 mg/g) was used as a standard.

### 4.5. Estimation of Total Phenolics

The total phenolic content was assessed with minor modifications, according to the method outlined by Johari and Khong (2019) [76]. A test tube was filled with 100 µL of AMFE and 500 µL of Folin–Ciocalteu reagent, which were mixed together and diluted with 6 mL of D/W. After the mixture had been mixed, it was left at 37 °C for 5 min. After that, 1.5 mL of 20% sodium carbonate (Na_2_CO_3_) was mixed in the tubes and the contents were gently shaken to ensure that the contents had been mixed well. After 90 min of mixing, the absorbance at 725 nm was measured using an ultraviolet-visible spectrophotometer. The standard reference was gallic acid, on the basis of which a calibration curve was prepared.

### 4.6. LC-MS Analysis

To analyze the phytochemical composition of AMFE, the HR-LCMS1290 Infinity UHPLC System with a PDA detector (Agilent Technologies, Santa Clara, CA, USA) was used. The system included a column compartment, quadrupole Time-of-Flight Mass Spectrometer (Q-TOF MS) with Agilent Jet Stream Electrospray ions, HiP sampler, and a binary gradient solvent pump. In the SB-C18 column (2.1 × 50 mm, 1.8 µm particle size), separation was performed. At a flow rate of 0.350 mL/min, 1% formic acid in deionized water (solvent A) and acetonitrile (solvent B) served as the mobile phase. Compound identification was based on the fragmentation patterns and mass spectra obtained from Q-TOF MS. Phytochemical constituents were identified using databases such as Compound Discoverer 2.1, ChemSpider, and PubChem [77].

### 4.7. Antioxidant Assay

#### 4.7.1. DPPH Assay

Antiradical activity was evaluated using a DPPH assay, following the procedure outlined via Brand-Williams et al. (1999) [78]. To prepare a fresh DPPH solution, 7.8 mg of DPPH was added in (20 mL) and freshly used. The assay involved mixing 1 mL of 0.1 mM DPPH with 100 μL of AMFE at varying solutions (20–100 μg/mL). After incubating for 15 min in a dark condition, the absorbance was measured at 517 nm. As a standard, quercetin was used and the percentage inhibition was calculated using the following formula.

Inhibition of DPPH radical (%) = Ab − As/Ab × 100

#### 4.7.2. FRAP (Ferric-Reducing Antioxidant Power) Assay

According to Benzie and Strain (1996) [79], the antioxidant capacity of AMFE was further assessed via the FRAP assay. The assay mixture was created by mixing 30 mM ferric chloride hexahydrate (FeCl_3_·6H_2_O), 10 mM TPTZ (2,4,6-tripyridyl-s-triazine), and 150 mM acetate buffer (pH—3.6) in 40 mM hydrochloric acid at a ratio of 10:1:1 at room temperature. Then, 3.95 mL of freshly made FRAP reagent was added to this combination along with 5 μL of various concentrations (20–100 μg/mL). After that, a 30 min room-temperature incubation period was observed. A blue-colored ferrous TPTZ complex was developed during this period as a result of ferric ions being reduced to ferrous ions in the presence of AMFE. Lastly, at 593 nm, the solution’s absorbance was measured.

### 4.8. AChE Inhibition

The AChE inhibition activity was assessed via the Ellman spectrophotometric method [80]. In the test, 3 mL of 0.1 M Tris-HCl buffer (pH 8.0) was mixed with 20 μL of AChE solution (3 U/mL). After adding 100 μL of AMFE at different doses (20–100 μg/mL) to this mixture, it was incubated for 15 min at room temperature. This was followed by the addition of 50 μL of 3 mM DTNB. The reaction was started by adding 50 μL of 15 mM acetylthiocholine iodide (AChI) as soon as a yellow color appeared. As a positive control, galantamine (1 mg/mL) was used. The solution’s absorbance was determined at 412 nm using a UV-visible spectrophotometer (UV-2600, Shimadzu, Japan). Based on a comparison of enzyme activity with a negative control, the following equation was used to calculate the percentage inhibition.

Inhibition (%) = A_0_ − A_1_ × 100

### 4.9. Thioflavin T (ThT) Assay

This method is widely employed to assess the rate of fibrillogenesis associated with amyloid development. ThT emits fluorescence upon binding to amyloid fibrils, making it an effective tool for monitoring in vitro amyloid fibril aggregation [81]. In this study, different concentrations of AMFE were combined with 20 μM of Aβ_1–42_ in a volume of 40 μL and incubated at 37 °C for 24 h. Afterwards, 100 μL of ThT solution was added to the mixture. As part of the analysis, the fluorescence intensities of the samples were measured using a FP-6200 spectrofluorometer (Shimadzu, RF-5000). Measurements were taken following a 30 min incubation with the excitation and emission wavelengths set at 450 nm and 483 nm, respectively. A positive control consisting of galantamine in combination with Aβ_1–42_ was included for comparison.

### 4.10. Neuroprotective Effects of the AMFE

#### 4.10.1. Preparation of AMFE Stock Solution

Stock solutions of the test samples were prepared in culture media at 1000 µg/mL and filtered through a 0.2-micron PES membrane syringe filter. In different wells, different concentrations of this solution were added to attain a 15.625–500 µg/mL concentration.

#### 4.10.2. Aβ_1–42_ Stock Solution and Working Solution

The Aβ_1–42_ was dissolved in D/W to a concentration of 1 mM. Treatments were conducted using a working solution of 100 μM in cell culture medium containing 10% FBS. After adding this solution to each well, a final concentration of 0.625–20 µM was achieved.

#### 4.10.3. Cell Culture

Using human SH-SY5Y neuroblastoma cells from the National Center for Cell Science in Pune, India, these studies were conducted. The DMEM medium containing non-essential amino acids (1X NEAA) and 10% Fetal Bovine Serum (FBS) was used for the growth of cells in a 5% CO_2_ condition at 37 °C. When the cells reached 80% confluence, experiments were conducted.

#### 4.10.4. Assessment of Cell Viability

Cell viability was evaluated via a modified MTT assay, as previously detailed [75], with all experiments conducted in triplicate. Cells grown in T-25 flasks were harvested by trypsinization and transferred into a 5 mL centrifuge tube. The cell pellet was collected by centrifuging at 300× *g*, and the resulting cells were resuspended in DMEM-HG medium. The cell concentration was adjusted to ensure that 200 μL of the suspension contained approximately 15,000 cells. A suspension of cells (200 μL per well) was placed into a 96-well microtiter plate and incubated at 37 °C in a humidified environment with 5% CO_2_ for 24 h. Following this incubation, the medium was gently removed and 200 μL of varying concentrations of AMFE was added to the designated wells. After that, the plate was put back in the incubator for a further 24 h period under the same settings. Following the second incubation, the medium was delicately removed, and 200 μL of new medium containing 10% MTT reagent was added to each well, resulting in a final MTT concentration of 0.5 mg/mL. The plate was incubated at 37 °C with 5% CO_2_ for three hours. The media was once more removed following incubation, leaving the MTT crystals undisturbed. Each well was added with the 100 μL of DMSO to dissolve the formazan crystals, and the plate was then well mixed by placing it on a gyratory shaker. Using a microplate reader, absorbance at 570 and 630 nm was measured to determine the viability of the cells. Background absorbance was subtracted and the percentage of growth inhibition was calculated. The IC_50_ value, indicating the concentration of the test compound required to reduce cell growth by 50%, was determined from the dose–response curve for the cell line.

#### 4.10.5. Assessment of Cytoprotective Activity

The protective effect of AMFE against Aβ_1–42_-induced toxicity was evaluated via the MTT assay, as previously outlined [82]. Cells were seeded into a 96-well plate and incubated for 24 h under the previously mentioned conditions. After this period, the medium was carefully aspirated from the wells. A volume of 100 μL of varying concentrations of AMFE was then added to the designated wells and incubated at 37 °C in a 5% CO_2_ atmosphere for 4 h. After this treatment, 100 μL of 10 μM and 20 μM β-Amyloid (prepared as a 2X solution) was supplemented to the respective wells, and the plate was incubated again under the same conditions for a period of 48 h. The media was taken out of each well after the incubation was finished, and 200 μL of new medium containing 10% MTT reagent was added, bringing the final MTT concentration to 0.5 mg/mL. The plate was incubated in 5% CO_2_ for three hours at 37 °C. The media was cautiously extracted after incubation, preserving the MTT crystals. In order to completely dissolve the formazan crystals, 100 μL of DMSO was added to each well, and the plate was gently shaken on a gyratory shaker. The absorbance was determined with a microplate reader at 570 and 630 nm.

#### 4.10.6. Mitochondrial Membrane Potential (ΔCm) Assay

First, cells were plated into 6-well plates at a density of 3 × 10^5^ cells per 2 mL. They were then left to adhere overnight at 37 °C in a CO_2_ incubator. The cells were cultured for a further 4 h after the culture medium was replaced with 2 mL of media containing 62.5 µg/mL of AMFE after 24 h. After that, the cells were exposed to 20 μM of Aβ_1–42_ and incubated for an additional 96 h. At room temperature, the cells were collected after the treatment by centrifuging them at 300× *g* for 5 min. After discarding the supernatant, the pellet underwent two PBS washes. After that, the cell pellet was again suspended in 0.5 mL of the freshly produced JC-1 working solution. To ensure uniform suspension, the cells were gently mixed using a pipette. After incubation in JC-1 solution for 10–15 min at 37 °C in a CO_2_ incubator, the cells were washed with 1X assay buffer, resuspended and immediately subjected to flow cytometric analysis [83,84].

#### 4.10.7. ROS Estimation by H_2_DCFDA Staining

At a density of 3 × 10^5^ cells per 2 mL, cells were plated in 6-well plates and incubated for 24 h at 37 °C in a CO_2_ incubator. The cells were treated with 62.5 µg/mL of AMFE in 2 mL of fresh culture media for 4 h after this initial incubation. After that, the cells were cultured for an extra 96 h at a concentration of 20 μM Aβ_1–42_. After the incubation period ended, each well was provided with 5 μL of 10 μM H_2_DCFDA, and the plate was incubated for 1 h at 37 °C. Following the incubation period, the cells were separated by trypsinization and gathered into 5 mL tubes in preparation for flow cytometric examination. The cells were pelleted by centrifugation at 300× *g* for 5 min at room temperature, the supernatant was removed, and the cells were washed twice with PBS. The final cell pellet was resuspended in 500 μL of pre-warmed DPBS and flow cytometry analysis was conducted with an excitation wavelength of 488 nm and detection at 525 nm (FL1) [85,86].

#### 4.10.8. Quantification of mRNA Using RT-qPCR

Cells were seeded and treated in 6-well plates as per the previously described method. Total RNA extraction was carried out using the Tri-Pure Isolation Reagent (Sigma-Aldrich^®^, Bangalore, India), according to the manufacturer’s protocol. RNA purity and concentration were measured using a NanoDrop Lite spectrophotometer (Thermo Scientific, Waltham, MA, USA), while RNA quality and integrity were assessed through denaturing agarose gel electrophoresis. Reverse transcription was performed with the RT First Strand Synthesis Kit (Qiagen, Valencia, CA, USA). The quantification of gene expression was achieved using SYBR Green-based quantitative real-time PCR (qRT-PCR) on the Applied Biosystems^®^ 7500 Fast Real-Time PCR system (Foster City, CA, USA). The qPCR procedure included an initial 2 min incubation at 50 °C, followed by denaturation at 95 °C for 5 min, and then 45 amplification cycles (20 s at 95 °C, 30 s at 60 °C, and 20 s at 72 °C). After amplification, a melt curve analysis was performed to confirm specificity, with the temperature increased from 50 °C to 98 °C, holding for 5 s at each 0.5 °C step. The reaction efficiency was evaluated using Lin-RegPCR software [87,88]. GAPDH served as the reference gene and the target genes analyzed included ACHE, APOE, GSK3β, and MAPT.

## 5. Conclusions

In conclusion, the present study demonstrates that AMFE exhibits significant neuroprotective potential towards the toxicity produced by Aβ_1–42_ in SH-SY5Y cells. Its capacity to block AChE activity, reduce ROS production, and restore MMP suggests that AMFE targets multiple pathways involved in AD pathogenesis. These results highlight the promise of AMFE as a natural treatment option for AD, warranting further validation through in vivo studies. Future research should focus on the determination of AMFE efficacy using relevant animal models, such as transgenic mice expressing human amyloid precursor protein and presenilin 1 mutations, to check its impact on AD-related neuropathology and cognitive decline. Other important parameters including optimal dosages, administration routes, and long-term effects should be determined to ensure translational relevance. Moreover, well-designed clinical trials are also essential to check the safety and efficacy of AMFE in AD patients, incorporating comprehensive evaluations of cognitive function, AD biomarkers, and the quality of life. Investigating the potential synergistic effects of AMFE with existing AD therapies, such as cholinesterase inhibitors or memantine, could provide valuable insights.

## Figures and Tables

**Table 1:** List of compounds identified from AMFE in positive mode of analysis via HR-LCMS. The table includes the compound name, molecular formula, mass and mass-to-charge ratio (*m*/*z*) values, and respective retention time (RT).

Compound Name	Formula	Mass	*m*/*z*	Polarity	RT
Rhazidigenine Nb-oxide	C_19_H_26_N_2_O_2_	314.1989	353.1618	Positive	2.267
Mukonicine	C_20_H_21_NO_3_	323.1522	328.1309	Positive	3.356
Benzosimuline	C_20_H_19_NO_2_	305.1419	310.1205	Positive	3.659
Vincamine	C_21_H_26_N_2_O_3_	354.1938	359.1726	Positive	4.181
Falcarindione	C_17_H_20_O_2_	256.1477	239.1445	Positive	5.001
L-alpha-Aspartyl-L-hydroxyproline	C_9_H_14_N_2_O_6_	246.0846	269.0737	Positive	9.546
5,6-Dihydrouridine	C_9_H_14_N_2_O_6_	246.0852	269.0744	Positive	9.9
6-Gingesulfonic acid	C_17_H_26_O_6_S	358.1444	341.1408	Positive	10.133
Knipholone	C_24_H_18_O_8_	434.101	417.0978	Positive	10.702
Erinacine C	C_25_H_38_O_6_	434.2682	417.2649	Positive	10.843
N1,N5,N10-Triferuloyl spermidine	C_37_H_43_N_3_O_9_	672.2932	655.2898	Positive	10.951
Austalide K	C_25_H_32_O_5_	412.2264	395.2231	Positive	11.032
Neamine (Neomycin A)	C_12_H_26_N_4_O_6_	322.1834	327.162	Positive	11.401
9-[(3,7-Dimethyl-2,6-octadienyl)oxy]-7H-furo [3,2-g][1]benzopyran-7-one	C_21_H_22_O_4_	338.154	321.1513	Positive	11.626
Apo-8′-lycopenal	C_30_H_40_O	416.3142	421.2928	Positive	12.13
1-Hydroxy-3,5-dimethoxy-2-prenylxanthone	C_20_H_20_O_5_	340.1337	323.1305	Positive	12.599
6-Gingesulfonic acid	C_17_H_26_O_6_S	358.1441	341.1408	Positive	12.599
Carindone	C_31_H_44_O_6_	512.3137	535.3051	Positive	13.089
6-Hydroxyl-1,6-dihydropurine ribonucleoside	C_10_H_14_N_4_O_5_	270.0933	253.09	Positive	13.19
L-alpha-Aspartyl-L-hydroxyproline	C_9_H_14_N_2_O_6_	246.0841	269.0733	Positive	13.194
1-Hydroxy-3,5-dimethoxy-2-prenylxanthone	C_20_H_20_O_5_	340.134	323.1309	Positive	14.211
Fargesone A	C_21_H_24_O_6_	372.1592	355.1559	Positive	14.309
Threoninyl-glutamate	C_9_H_16_N_2_O_6_	248.0995	271.0887	Positive	14.484
(+/−)-5-Deoxykievitone	C_20_H_20_O_5_	340.1334	323.1301	Positive	14.577
Goshonoside F6	C_31_H_52_O_12_	616.3433	617.3462	Positive	14.953
Phytolaccinic acid	C_31_H_48_O_6_	516.3472	555.3102	Positive	15.406
3′-N-Acetyl-4′-O-(10,12-octadecadienoyl)fusarochromanone	C_35_H_52_N_2_O_6_	596.3775	601.3525	Positive	16.162
Garcinol	C_38_H_50_O_6_	602.3618	603.3701	Positive	16.321
Janthitrem E	C_37_H_49_NO_6_	602.3606	603.3682	Positive	16.585
Eudesobovatol A	C_33_H_44_O_4_	504.3259	527.3151	Positive	16.813
Rescinnamine	C_35_H_42_N_2_O_9_	634.2932	617.2909	Positive	17.7
Flavidulol C	C_34_H_42_O_4_	514.3101	537.2993	Positive	18.151
3-O-trans-Feruloyleuscaphic acid	C_40_H_56_O_8_	664.3989	685.3513	Positive	18.303
Avadharidine	C_36_H_51_N_3_O_10_	684.3439	685.3509	Positive	18.475
Prosapogenin	C_36_H_54_O_11_	662.3626	667.3412	Positive	18.501

**Table 2:** List of compounds identified from AMFE in negative mode of analysis via HR-LCMS. The table includes the compound name, molecular formula, mass and mass-to-charge ratio (*m*/*z*) values, and respective retention time (RT).

Compound Name	Formula	Mass	*m*/*z*	RT
Quinic acid	C_7_H_12_O_6_	192.0633	191.0561	1.351
Benzoic acid	C_7_H_6_O_2_	122.0367	121.0298	16.145
m-Coumaric acid	C_9_H_8_O_3_	164.0476	163.0401	7.556
Quercitrin	C_21_H_20_O_11_	448.102	447.0948	8.325
Maritimetin	C_15_H_10_O_6_	286.0468	285.0395	11.816
Genistein	C_15_H_10_O_5_	270.0533	315.0514	12.041
4-Hydroxy-3-methoxy-2,10-bisaboladien-9-one	C_16_H_26_O_3_	266.1891	265.1818	22.224
Oleamide	C_18_H_35_NO	281.2731	340.2873	22.114
Gallic acid	C_7_H_6_O_5_	170.0221	169.0148	3.126
Caffeic acid	C_9_H_8_O_4_	180.0429	179.0356	6.409
Benzoic acid	C_7_H_6_O_2_	122.0367	121.0298	16.145
Vanillic acid	C_8_H_8_O_4_	168.0415	167.0348	7.556
Chlorogenic acid	C_16_H_18_O_9_	354.0965	353.0893	4.815
(-)-Epicatechin	C_15_H_14_O_6_	290.0805	289.0733	12.041
Kakuol	C_10_H_10_O_4_	194.0586	193.0513	9.427
Ellagic acid	C_14_H_6_O_8_	302.0074	301.0002	7.923
2,6-dihydroxybenzoic acid	C_7_H_6_O_4_	154.0272	153.0199	4.607
L-Malic acid	C_4_H_6_O_5_	134.022	133.0147	1.757
Sterculic acid	C_19_H_34_O_2_	294.2563	293.2488	24.507
Neocarthamin	C_21_H_22_O_11_	450.1185	449.1113	6.6
2-O-Caffeoylarbutin	C_21_H_22_O_10_	434.1232	433.1161	7.547
Garciduol C	C_27_H_18_O_9_	486.0978	485.0904	12.194
Kawain	C_14_H_14_O_3_	230.0955	229.0882	13.389
Piscerythramine	C_26_H_29_NO_6_	451.2025	450.1955	16.1
Pyropheophorbide a	C_33_H_34_N_4_O_3_	534.2627	593.2766	16.794
Pateamine	C_31_H_45_N_3_O_4_S	555.3203	614.3344	17.472
alpha-licanic acid	C_18_H_28_O_3_	292.2057	291.1984	17.572
Salannin	C_34_H_44_O_9_	596.2997	595.2923	18.756
dolichyl diphosphate	C_25_H_46_O_7_P_2_	520.2737	579.2881	19.05
2-[4,6-Bis(2,4-dimethylphenyl)-1,3,5-triazin-2-yl]-5-(octyloxy)phenol	C_33_H_39_N_3_O_2_	509.3019	554.3057	19.93
Cucurbitacin E	C_32_H_44_O_8_	556.3015	555.2933	20.096
Corchorosol A	C_29_H_44_O_9_	536.305	581.3036	21.609
α-Linolenic Acid	C_18_H_30_O_2_	278.2275	277.2204	22.149
Linalyl caprylate	C_18_H_32_O_2_	280.2432	279.2362	23.497
Palmitic Acid	C_16_H_32_O_2_	256.2419	255.2347	24.606
omega-hydroxy behenic	C_22_H_44_O_3_	356.3316	355.3243	24.771
Palmitic Acid	C_16_H_32_O_2_	256.2421	255.2348	24.942
Petroselinic acid	C_18_H_34_O_2_	282.2582	281.251	25.13
1,2,10-Trihydroxydihydro-trans-linalyl oxide 7-O-beta-D-glucopyranoside	C_16_H_30_O_10_	382.1846	381.1777	25.131

## Data Availability

All data are included in this manuscript.
